# Optimized Rectifier Topologies for Low-Voltage Electromagnetic Energy Harvesters

**DOI:** 10.3390/s26061887

**Published:** 2026-03-17

**Authors:** Niklas Krug, Felix Heer, Gerhard Fischerauer

**Affiliations:** Chair of Measurement and Control Systems and Center of Energy Technology (ZET), University of Bayreuth, 95447 Bayreuth, Germany

**Keywords:** electromagnetic energy harvesting, low voltage, rectification, power conversion efficiency

## Abstract

Vibrational energy harvesters typically generate only low voltages and low powers, making high-efficiency power conversion essential to extract usable energy from such sources. To address this challenge, suitable rectifier circuits must be designed to operate efficiently under low-voltage conditions. In this study, three rectifier topologies—a standard bridge rectifier and two alternative designs from the literature—were investigated in a two-step methodology: first, measurements were performed in the laboratory using a function generator to simulate controlled excitation conditions, followed by experiments with a real electromagnetic energy harvester. Component-level testing allowed the identification of the most suitable components for each topology, highlighting the influence of parameters such as MOSFET gate-source threshold voltage on overall performance. Using the selected optimal components, the circuits were then compared under varying excitation amplitudes and load conditions. Small modifications were introduced to the literature designs to improve switching behavior and reduce conduction losses. Across all tested conditions, the active-diode rectifier consistently achieved the highest harvested power, demonstrating both the effectiveness of component selection and the practical benefit of the adapted topology. These results provide a systematic basis for designing high-efficiency rectifiers for low-voltage vibrational energy harvesting applications.

## 1. Introduction

In recent years, the use of wireless sensor nodes (WSNs) and Internet of Things (IoT) devices in industrial and infrastructure applications has increased significantly [1,2,3]. Since these systems are often operated in locations that are difficult to access, an independent power supply without regular battery replacement is required [4,5]. Ambient energy can be converted into electrical power using energy harvesting technologies, enabling virtually autonomous system operation. Electromagnetic energy harvesting is particularly suitable for applications involving continuous vibrations or movements, such as in machines, vehicles, or building structures [6,7,8,9].

Recent years have witnessed substantial progress in the practical implementation of vibration energy harvesting systems in engineering applications. Current research increasingly emphasizes system-level integration into intelligent monitoring platforms and self-powered sensing systems or networks. Representative works demonstrate the deployment of vibration energy harvesters in structural health monitoring, machine condition monitoring, and industrial IoT frameworks, highlighting the transition from laboratory prototypes toward autonomous sensing solutions under realistic operating conditions [10,11,12,13]. Within these application-oriented systems, the harvester forms part of a multi-stage energy conversion chain including rectification, power management, energy storage, and sensing modules. Consequently, overall system reliability and long-term autonomous operation depend not only on electromechanical energy conversion, but critically on the efficiency and start-up capability of the front-end rectification stage.

**Figure 1:**
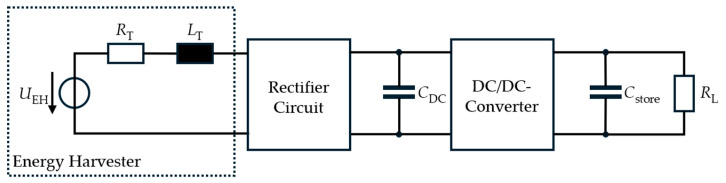
Typical structure of an electromagnetic energy harvesting system including rectification and DC/DC conversion.

Its operating principle follows the law of electromagnetic induction, where the relative motion between a coil and a magnet generates an electrical voltage. This induced voltage is quasi-stochastic and depends on the amplitude of movement, the magnetic field strength, and the coil geometry [14]. Figure 1 illustrates the fundamental structure of an electromagnetic energy harvesting system. The harvester can be electrically represented as an AC voltage source *U*_EH_(*t*) in series with an effective transducer resistance *R*_T_ and an effective transducer inductance *L*_T_. These components should not be interpreted as the coil’s intrinsic resistance or inductance alone. They also account for effects such as magnetic coupling, mechanical damping, and other system-level interactions, as detailed in [15]. The AC voltage generated by the harvester is subsequently rectified and temporarily stored in a buffer capacitor *C*_DC_. A downstream DC/DC converter amplifies the voltage to a usable level in order to supply consumers such as microcontrollers or radio units with the equivalent resistance *R*_L_ via a large storage capacitor *C*_store_ [16].

Since the induced voltage is typically only in the range of millivolts and only occasionally reaches a few volts, rectification poses a particular challenge [17,18]. The forward voltage of conventional diodes (typically 0.3–0.7 V) is often comparable to the signal level, resulting in significant energy losses or even preventing rectification altogether [19]. Therefore, the choice of a suitable rectifier circuit is crucial for the overall efficiency of the system.

Various alternative rectifier topologies have been proposed in the literature to minimize losses at low input voltages [19,20,21,22]. The aim of these approaches is to increase power output and improve energy conversion efficiency through the selection of suitable components and circuit architecture. In addition to classic diode bridges, MOSFET-based circuits, in which the intrinsic diodes of the transistors or actively controlled MOSFETs are used for rectification, are increasingly used [23]. Well-known concepts include the 4N-rectifier and circuits with active diodes that minimize forward losses via feedback control circuits [19,20,24,25,26,27,28]. Some approaches in the literature integrate the rectification stage directly with a boost converter to improve overall power transfer [29,30,31]. In this work, such combined architectures were not considered because a BQ25504 is already used downstream, which efficiently boosts the rectified voltage. Furthermore, the paper is intended to remain relevant for applications that focus solely on the rectification stage without requiring integrated step-up conversion. In addition, much of the published literature considers the circuits exclusively under theoretical or simulated conditions without verifying them on a real energy harvester setup. In our work, we have therefore investigated three rectifier circuits experimentally—a conventional bridge rectifier and a 2N2D rectifier (an optimized variant of the 4N rectifier) to analyze the performance of MOSFET-based rectification and an active-diode rectifier, selected as a representative of actively controlled circuits that have shown the most promising performance in the literature. The evaluation has been carried out both under controlled laboratory conditions and on a real electromagnetic energy harvester to assess the performance of the circuits in practical use.

## 2. Materials and Methods

### 2.1. Experimental Setup

#### 2.1.1. Preliminary Tests Under Controlled Source Conditions

**Figure 2:**
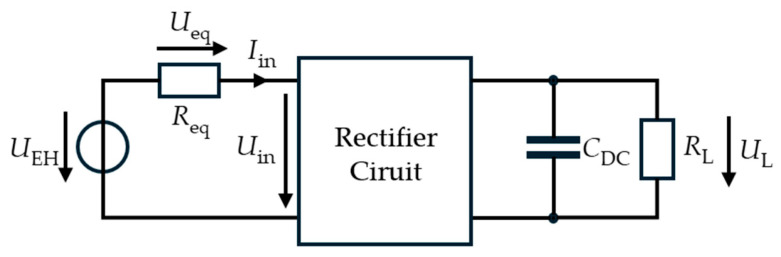
Equivalent circuit of the electromagnetic energy harvester used for experimental investigations. The harvester is represented as an AC voltage source with internal resistance *R*_eq_; the inductive impedance is neglected. The source drives the rectifier circuit, which charges the buffer capacitor *C*_DC_ and supplies the load resistance *R*_L_.

The experimental investigations were carried out in two parts. In the first part, the energy harvester was replaced by an AC voltage source to enable controlled measurement of the input current. The source was modeled with an equivalent resistance $R_{\mathrm{eq}}=1.5\;k\Omega$, which was experimentally determined by varying the connected load *R*_L_ and observing the resulting output power. This representation captures the real (active) power behavior, while the inductive effects, which introduce a phase shift between voltage and current, are not explicitly modeled. Since all measurements were performed at a single operating frequency, this approximation is sufficient for the analysis of power, see Figure 2.

The instantaneous output power is calculated by measuring the voltage over the load resistor *R*_L_

(1) $$P_{\mathrm{out}}=\frac{U_{L}^{2}}{R_{L}}.$$

The average output power $\bar{P_{\mathrm{out}}}$ is then obtained by averaging the instantaneous output power over a time period Δt:

(2) $$\bar{P_{\mathrm{out}}}=\frac{1}{\Delta t}\cdot \int_{t}^{t+\Delta t}\frac{U_{L}^{2}(\tau )}{R_{L}}d\tau .$$

The efficiency η is defined as the ratio of output energy to input energy and can therefore be calculated using the following equation:

(3) $$\eta =\frac{\bar{E_{\mathrm{out}}}}{\bar{E_{\mathrm{in}}}}=\frac{\int_{t}^{t+\Delta t}\frac{U_{L}^{2}(\tau )}{R_{L}}d\tau }{\int_{t}^{t+\Delta t}I_{\mathrm{in}}(\tau )\cdot U_{\mathrm{in}}(\tau )d\tau }=\frac{R_{\mathrm{eq}}}{R_{L}}\cdot \frac{\int_{t}^{t+\Delta t}U_{L}^{2}(\tau )}{\int_{t}^{t+\Delta t}U_{\mathrm{eq}}(\tau )\cdot (U_{\mathrm{EH}}(\tau )-U_{\mathrm{eq}}(\tau ))}d\tau$$

with $I_{\mathrm{in}}=\frac{U_{\mathrm{eq}}}{R_{\mathrm{eq}}}$ and $U_{\mathrm{in}}=U_{\mathrm{EH}}-U_{\mathrm{eq}}$.

A stimulation frequency of 25.5 Hz was selected for the entire experiment as it corresponds to the resonance frequency of the electromagnetic energy harvester described in the next section.

To quantify the dependence of the output power on the load, the load resistance *R*_L_ was varied. In the measurement, this replaces the DC/DC converter, which handles load matching in the real energy harvesting system. By varying *R*_L_, the optimal load point can be determined, and the performance and efficiency of the various rectifier circuits can be compared under the same conditions.

#### 2.1.2. Experimental Tests Under Real Vibration Excitation

**Figure 3:**
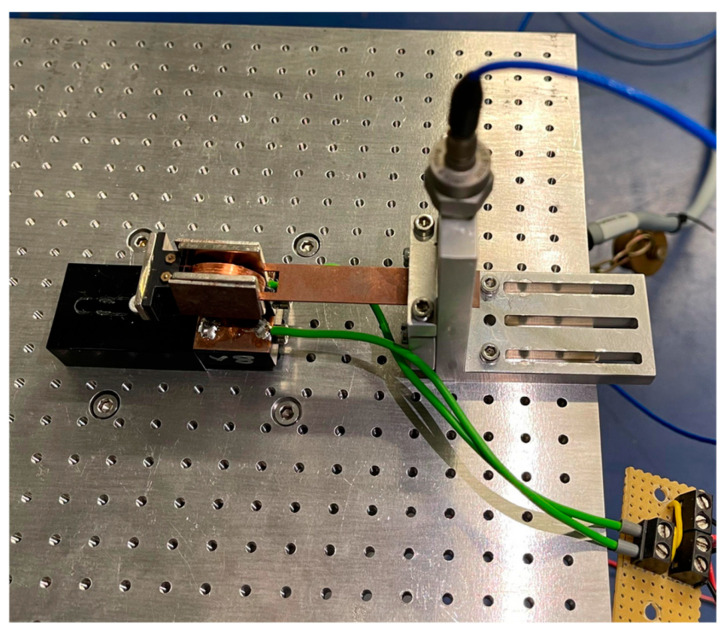
Experimental setup with the electromagnetic vibration energy harvester. The cantilever with attached magnets is excited via a shaker driven by a function generator and amplifier. The induced AC voltage in the fixed coil is used to supply the rectifier circuits under test.

Figure 3 shows a picture of the electromagnetic energy harvester used in this work, originally developed by Mösch [32,33]. The base plate is set into controlled vibration using a function generator (Agilent 33210A, Santa Clara, CA, USA) in combination with a power amplifier driving a shaker (B&K LDS V406, Sydney NSW, Australia). This excites the beam in resonance, so that a maximum vibration amplitude is achieved.

The induced voltage is generated by the relative movement of the magnets attached to the front and the coil fixed on the base. The rectifier circuits are connected to the ends of this coil to convert the quasi-stochastic voltage into direct voltage.

The output voltage of the rectifier is continuously measured via a data acquisition card. At the same time, the acceleration *a*(*t*) of the ground is recorded using an acceleration sensor (B&K 4534-B-001) to quantify the vibration forces acting on the harvester. The harvester is always operated at its resonance frequency of 25.5 Hz to achieve maximum power output. The measurements allow direct determination of power output and efficiency under real-world operating conditions. By combining output voltage and measured acceleration, the relationship between excitation amplitude, load, and energy yield can be analyzed.

### 2.2. Rectifier Topologies

**Figure 4:**
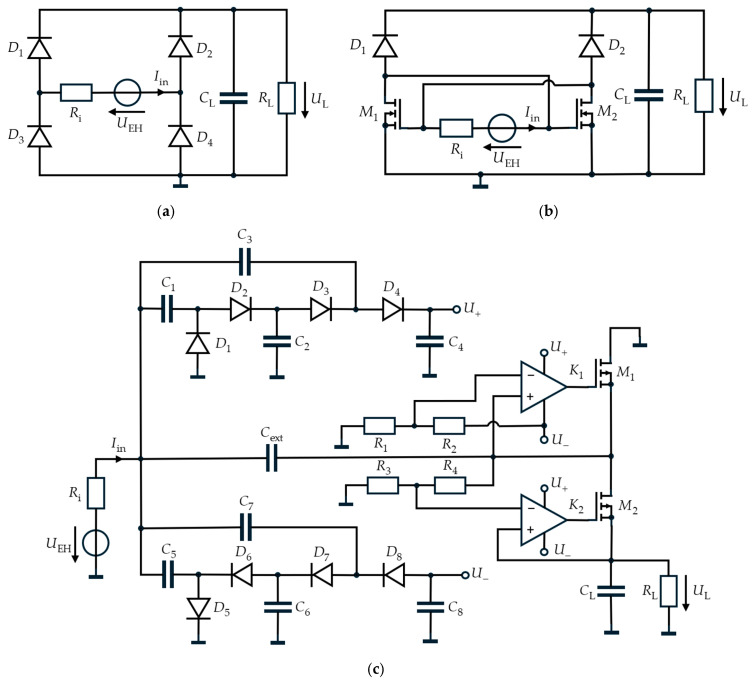
Circuit topologies of the investigated rectifier configurations: (**a**) full-bridge rectifier; (**b**) 2N2D-rectifier; and (**c**) active-diode rectifier.

The rectifier topologies investigated in this paper, shown in Figure 4, form the basis of the subsequent experimental analysis. These circuits represent three different approaches to AC-DC conversion in low-voltage energy harvesting systems.

#### 2.2.1. Full-Bridge Rectifier

The full-bridge rectifier is one of the most widely used rectifier configurations [21]. It consists of four diodes (*D*_1_–*D*_4_), with two conducting during each half-cycle while the remaining two are reverse-biased. This results in two significant loss mechanisms based on the forward voltage of the diodes. On the one hand, the current flow is delayed after a voltage zero crossing because the diodes must first overcome the forward voltage. Secondly, a continuous voltage drop occurs across the diodes even during conduction, which reduces the output voltage. In addition, a small reverse current flows during reverse operation, contributing to further losses.

#### 2.2.2. 2N2D Rectifier

The 2N2D rectifier is based on the 4N topology and resembles a passive bridge rectifier circuit [19]. It transmits both positive and negative half-waves, with the two MOSFETs *M*_1_ and *M*_2_ conducting at positive and negative input voltages, respectively. Unlike diodes, they switch with virtually no loss, as the forward voltage is almost zero, but only become active above the threshold voltage *U*th of the MOSFET. Compared to the 4N rectifier, the upper two MOSFETs were replaced by diodes *D*_1_ and *D*_2_, since they originally operated as diode-connected MOSFETs. As the selected diodes exhibit very low forward losses, it was found experimentally that using diodes in the upper branches leads to improved performance. Based on these observations, the modified topology, the 2N2D rectifier, will be considered.

#### 2.2.3. Active-Diode Rectifier

Unlike the passive topologies presented so far, the active-diode rectifier requires external control: MOSFETs *M*_1_ and *M*_2_ are switched selectively via the comparators *K*_1_ and *K*_2_, so that the forward voltage is almost eliminated. This enables a significantly higher output voltage and efficiency, especially at low input amplitudes. The circuit consists of two components, as described below [27].

In the first stage, the diodes *D*_1_–*D*_8_ and capacitors *C*_1_–*C*_8_ form a voltage cascade that converts the alternating input voltage *U*_EH_ into a positive and a negative DC voltage, *U*_+_ and *U*_−_, which reach approximately four times the input amplitude under open-circuit conditions. These voltages are used to supply the comparators. Although the cascade itself operates with relatively low efficiency, it is well suited for this purpose since the comparators draw only minimal current while the circuit provides sufficiently high and symmetric supply voltages.

**Figure 5:**
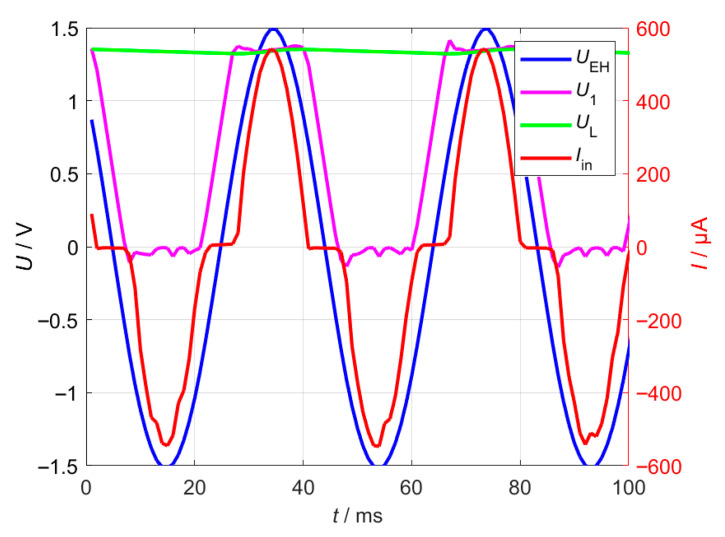
Voltages *U*_EH_, *U*_1_ and *U*_L_ and current *I*_in_ measured in the active-diode rectifier, showing the circuit’s dynamic response and switching behavior. The rectifier implementation, including the diodes, MOSFETs, and operational amplifier used, is summarized in Table 1.

To illustrate the operation of the remaining circuitry, the voltage waveforms *U*_EH_, *U*_1_ and *U*_L_ were recorded and plotted in Figure 5. Immediately after the input, an intermediate capacitor *C*_ext_ is placed to buffer the voltage, allowing its voltage to rise to approximately twice the input voltage amplitude under no-load conditions. The MOSFETs are controlled based on the output of the comparators:

- During the negative half-wave, comparator *K*_1_ detects that the voltage *U*_1_ is below the reference voltage and activates MOSFET *M*_1_, which only charges *C*_ext_. The load voltage *U*_L_ drops due to the connected load *R*_L_.
- In the positive half-cycle, the combination of the input voltage *U*_EH_ and the precharged capacitor *C*_ext_ enables efficient charging of the load capacitor *C*_L_. Under no-load conditions, the voltage *U*_L_ would rise to just below twice the input amplitude.

**Table 1:** Component selection and dimensioning for the rectifier circuits investigated.

Rectifier Circuit	Component	Designation	Value
Full-Bridge Rectifier	*D*_1_–*D*_4_	MBR30H30CTG [34]	
2N-2D Rectifier	*D*_1_, *D*_2_	MBR30H30CTG [34]	
	*M*_1_, *M*_2_	BSR802N L6327 [35]	
Active-Diode Rectifier	*D*_1_–*D*_8_	1SS422(TE85L,F) [36]	
	*C*_1_–*C*_8_		4.7 µF
	*M*_1_, *M*_2_	SQJ123ELP [37]	
	*K*_1_, *K*_2_	TLV8802DGKR [38]	
	*R*_1_, *R*_4_		10 kΩ
	*R*_2_, *R*_3_		1 MΩ
Load (all Circuits)	*C*_L_		100 µF

The reference voltage of comparator *K*_1_ was set slightly below 0 V, while the reference voltage of comparator *K*_2_ was set slightly below *U*_1_ using voltage dividers (*R*_1_ and *R*_2_ or *R*_3_ and *R*_4_). This arrangement prevents undesired switching states due to non-ideal comparator behavior and was confirmed experimentally.

#### 2.2.4. Component Selection and Dimensioning

The selection and dimensioning of the components were primarily guided by the characteristics of the devices. For the diodes in the full-bridge rectifier, the forward voltage drop was the main criterion, as lower forward voltages improve conduction efficiency. Among ten diodes tested, the MBR30H30CTG [34] was found to perform best and is also suitable for the 2N2D rectifier. However, these diodes proved unsuitable for use in the voltage cascade stage due to their relatively high reverse leakage current. Since this stage is lightly loaded, the reverse current significantly affects the operation. Therefore, diodes with lower reverse leakage, such as the 1SS422 [36], were chosen for the voltage cascade.

For the MOSFET selection in the 2N2D rectifier, a low gate-source threshold voltage *U*th was the most critical criterion. The BSR802NL6327 [35] exhibited the lowest threshold voltage among the devices tested and was therefore chosen. In the active-diode rectifier, the key considerations for the p-type MOSFETs are a minimal on-resistance *R*_DS,on_ at the lowest possible threshold voltage, as well as a low gate capacitance to reduce charge losses during switching, thereby minimizing the voltage drop across the MOSFETs during conduction.

For the comparators, low power consumption is a primary requirement. In addition, the comparators should exhibit fast switching behavior and a minimal offset voltage to ensure accurate and efficient operation of the active-diode rectifier. Table 1 provides an overview of the component selection and their corresponding values.

## 3. Results

### 3.1. Load-Dependent Performance

**Figure 6:**
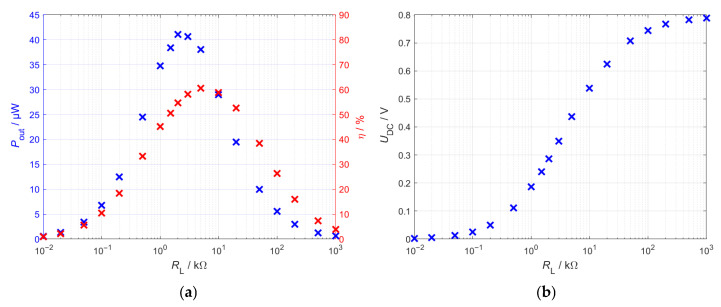
Characteristics of the bridge rectifier at an input voltage amplitude of ${\hat{U}}_{\mathrm{EH}}=1\;V$, obtained by varying the load resistance. (**a**) Output power *P*_out_ and efficiency η; (**b**) corresponding DC output voltage *U*_DC_.

To analyze the load behavior, the input amplitude ${\hat{U}}_{\mathrm{EH}}$ was set to 1 V while the load resistance *R*_L_ was varied. Figure 6a shows the average power and efficiency plotted against the load resistance for the full-bridge rectifier by way of an example. The load resistance at which power becomes maximum defines the maximum power point (MPP) of the system. The efficiency also reaches its maximum at a certain load resistance. However, this is slightly above the load resistance in the MPP. Thus, the efficiency does not reach its maximum in the MPP. Nevertheless, the MPP is of central importance for the energy harvesting system as this is where the maximum possible power is obtained from the system [39].

High efficiency is particularly important in weak environments, where “weak” refers to systems with a small proof mass. In such systems, energy extraction by the harvester leads to significant damping, so low-efficiency rectification would further damp the system by reducing the mechanical motion.

Figure 6b illustrates that the DC output voltage increases with rising load resistance. At the load resistance *R*_MPP_, the DC voltage *U*_DC_ reaches approximately half of its open-circuit value. Consequently, the downstream DC/DC converter must regulate its input voltage to roughly half of the open-circuit voltage in order to ensure that the rectifier stage operates continuously at the maximum power point (MPP), thereby enabling maximum power transfer from the system.

**Figure 7:**
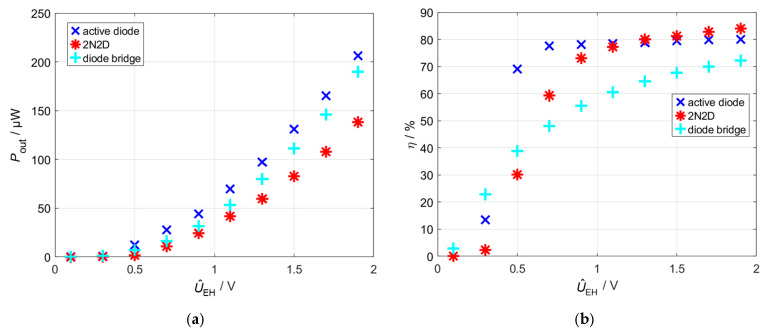

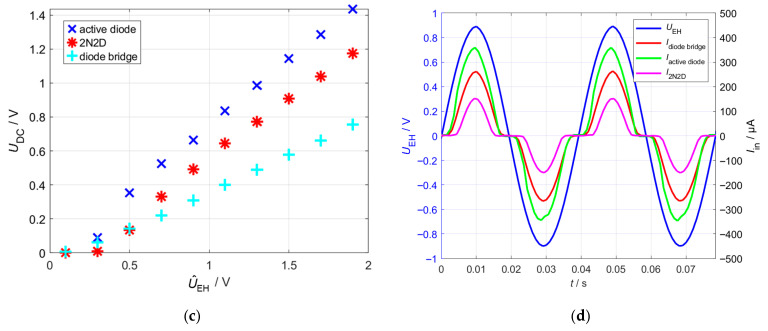
Measured performance of the three circuits at varying input voltage amplitudes. (**a**) Output power; (**b**) efficiency; (**c**) output DC voltage; and (**d**) load current waveforms.

To compare the three rectifier circuits under investigation, the amplitude ${\hat{U}}_{\mathrm{EH}}$ was varied on the frequency generator while the circuits were operated in MPP. Figure 7a shows the measured power and efficiency characteristics of the three rectifier topologies. The active-diode rectifier achieves the highest output power, followed by the bridge rectifier. The 2N2D rectifier exhibits slightly lower output power levels compared to the other two configurations. When considering efficiency, a more nuanced picture emerges: At low input amplitudes, the active-diode rectifier achieves the highest efficiency. However, at input amplitudes of around 1 V or higher, the 2N2D rectifier outperforms it as its switching losses are less significant at higher voltages. With the bridge rectifier, efficiency increases continuously with increasing input voltage but remains below the values of the other two topologies, except in the range of very low voltages.

The efficiency of the active-diode rectifier not only reflects the rectification stage itself but also represents the overall system efficiency. This is because the comparators are powered directly by the voltage cascade, and their power consumption is therefore inherently included in the measured output. As a result, the reported efficiency values provide a true net energy conversion performance for the entire rectifier system. The results can also be explained by the current curves shown in Figure 7d. The 2N2D rectifier switches very late when a positive half-wave arrives. This is due to the threshold voltage *U*th of the MOSFETs *M*_1_ and *M*_2_. It can also be seen that the voltage drop across the MOSFETs is higher than across the optimally chosen diodes. A possible explanation is that the MOSFET does not sufficiently conduct at this point, leading to an increased *R*_DS,on_. The circuit is therefore totally dependent on the selection of diodes. The current through the bridge rectifier is higher than that of the 2N2D rectifier. Overall, the results indicate that the active-diode rectifier initiates conduction earlier and incurs a significantly reduced voltage drop across its components.

When operating with a downstream DC/DC converter, the maximum power is not always hit exactly, which can slightly reduce the overall system efficiency; however, this effect applies equally to all rectifier circuits examined, so that the relative efficiency differences remain unchanged.

### 3.2. Performance Evaluation on the Real Electromagnetic Energy Harvester

**Figure 8:**
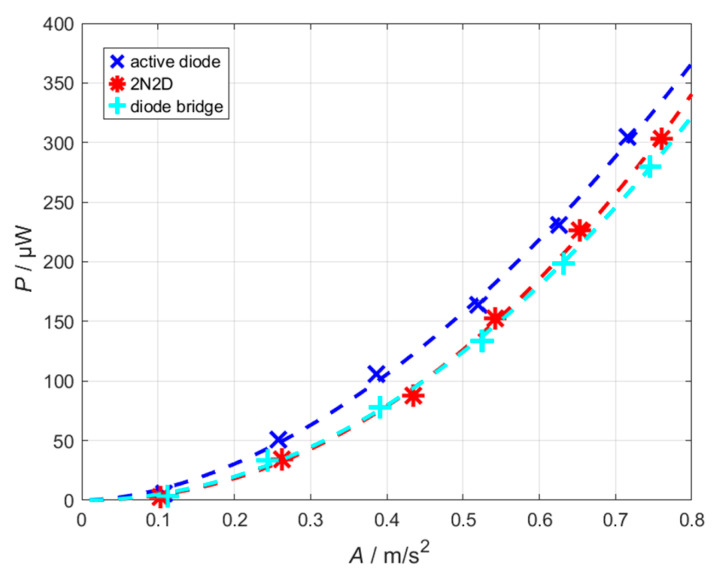
Measured output power of the energy harvester for different acceleration amplitudes using the three evaluated rectifier topologies. Dashed lines in corresponding colors indicate fits using power functions to the data, providing a quantitative approximation of the performance.

To validate the results on the real system, the three circuits were tested on the electromagnetic energy harvester. It should be noted that the set voltages on the connected function generator do not reproducibly deliver the same acceleration amplitudes. For this reason, the acceleration amplitude was always measured, and the power output was plotted against it. Figure 8 shows the measured results, including power-function fits for quantitative comparison The active-diode rectifier delivers the highest power across the entire acceleration range. The 2N2D-rectifier shows surprisingly strong performance, which may indicate that efficiency plays a more significant role in the real system than initially expected. Using the power-function fits, it can be determined that at an acceleration amplitude of 0.5 m/s^2^, the output power of the active-diode rectifier is approximately 30 µW higher than that of the other two rectifier circuits, corresponding to a power increase of about 25%.

The extracted fit-parameters of the applied power-law model are summarized in Table 2, including their associated 95% confidence intervals. Among the evaluated rectifier topologies, the active-diode rectifier exhibits the highest value of *x*_1_, indicating the most favorable performance at low acceleration amplitudes. This observation is consistent with the measured data in the low-acceleration area. However, the power-law fits listed in Table 2 further reveal a steeper scaling behavior for the 2N2D rectifier, suggesting that, when extrapolating the fitted model toward higher acceleration amplitudes, this topology is expected to deliver a higher output power compared to the active-diode rectifier.

Under weak-environment conditions, characterized by very small vibration amplitudes, the active-diode rectifier is particularly suitable due to its low MOSFET threshold voltage, which allows earlier conduction and efficient rectification. A key limitation arises from the supply of the comparators via the voltage cascade: if the comparator supply voltage is too low, the switches do not operate, and the rectifier delivers no output. In this case, the net harvested power is zero, but not negative, since the system is fully self-powered. At moderate vibration levels, it is evident from the measurements that the active-diode rectifier achieves the highest power output compared to the other topologies.

Although all experiments were conducted at the resonance frequency of the electromagnetic energy harvester to ensure maximum and reproducible performance conditions, frequency deviations from resonance often occur in practical applications. Under non-resonant excitation, the mechanical vibration amplitude of the system is reduced, resulting in a lower induced voltage and thus reduced input power at the rectifier. This operating state is functionally comparable to operation at a reduced acceleration amplitude, as investigated in Figure 8. The results shown there indicate that the active-diode rectifier achieves the highest output power value even at small acceleration amplitudes. It can therefore be expected that the active-diode rectifier will also exhibit advantageous performance characteristics under non-resonant operating conditions. In addition, there exist methods that adjust the resonance frequency of a harvester to the excitation frequency if the frequency difference is sufficiently small [32].

## 4. Conclusions

In this work, a standard bridge rectifier was systematically compared with two alternative topologies from literature. Measurements were first conducted using a function generator and subsequently on an actual electromagnetic energy harvester. The results demonstrate a strong load-dependent behavior of all rectifier circuits. To ensure optimal energy extraction, a downstream DC/DC converter must continuously monitor the open-circuit voltage of the harvester and adjust its input voltage to approximately half of this value, effectively emulating the optimal load impedance. Furthermore, the performance of each topology is highly sensitive to component selection. In particular, the 2N2D rectifier shows potential for improvement, as its performance is strongly influenced by the gate-source threshold voltage of the MOSFETs. A lower threshold enables earlier switching, reduces voltage drop during conduction and leads to higher harvested power.

Overall, the active-diode rectifier consistently delivered the highest power across all excitation amplitudes, making it the most suitable choice for such low voltage energy harvesting applications like electromagnetic energy harvesters. However, efficiency remains an important factor, as higher efficiency reduces the damping of the source and allows more energy to be extracted. Measurements on the real system further confirmed that the active-diode rectifier delivers the best performance, enabling an increase in harvested power on the order of several tens of percent in harvested power compared to the other two rectifier circuits. This underscores the practical advantage and effectiveness of such a system. This demonstrates that careful topology selection, combined with appropriate component choice and adaptive DC/DC regulation, is critical for maximizing energy extraction from small-scale vibrational sources.

The results of the investigated rectifier topologies may be sensitive to device tolerances. The paper identifies the key parameters—particularly the MOSFET threshold voltage—that have a significant impact on efficiency. In the future, devices with improved characteristics, such as lower threshold voltages, may become available. The presented results therefore provide a clear basis for estimating how the circuit-performance could be enhanced using alternative components.

## Figures and Tables

**Table 2:** Power-law fit-parameters *x*_1_ and *x*_2_ of $P(a)=x_{1}\cdot a^{x_{2}}$ with 95% confidence intervals for the evaluated rectifier topologies.

Rectifier Circuit	*x*_1_	*x*_2_
Full-Bridge Rectifier	504.8±31.4	2.018±0.136
2N-2D Rectifier	545.6±41.0	2.112±0.175
Active-Diode Rectifier	546.8±56.2	1.795±0.202

## Data Availability

The raw data supporting the conclusions of this article will be made available by the authors on request.
